# Large Positive Magnetoconductance in Carbon Nanoscrolls

**DOI:** 10.1021/acs.nanolett.4c03694

**Published:** 2025-03-28

**Authors:** Yu-Jie Zhong, Jia-Cheng Li, Xuan-Fu Huang, Ying-Je Lee, Ting-Zhen Chen, Jia-Ren Zhang, Angus Huang, Hsiu-Chuan Hsu, Carmine Ortix, Ching-Hao Chang

**Affiliations:** † Department of Physics, National Cheng Kung University, Tainan 70101, Taiwan; ‡ Center for Quantum Frontiers of Research and Technology (QFort), National Cheng Kung University, Tainan 70101, Taiwan; ¶ Program on Key Materials, Academy of Innovative Semiconductor and Sustainable Manufacturing, National Cheng Kung University, Tainan 70101, Taiwan; § Department of Physics, National Tsing Hua University, Hsinchu 30013, Taiwan; ∥ Graduate Institute of Applied Physics, National Chengchi University, Taipei 11605, Taiwan; ⊥ Dipartimento di Fisica “E. R. Caianiello”, Università di Salerno I-84084 Fisciano (Salerno), Italy

**Keywords:** radial superlattice, Aharonov−Bohm effect, interfacial magnetic states, longitudinal magnetic field

## Abstract

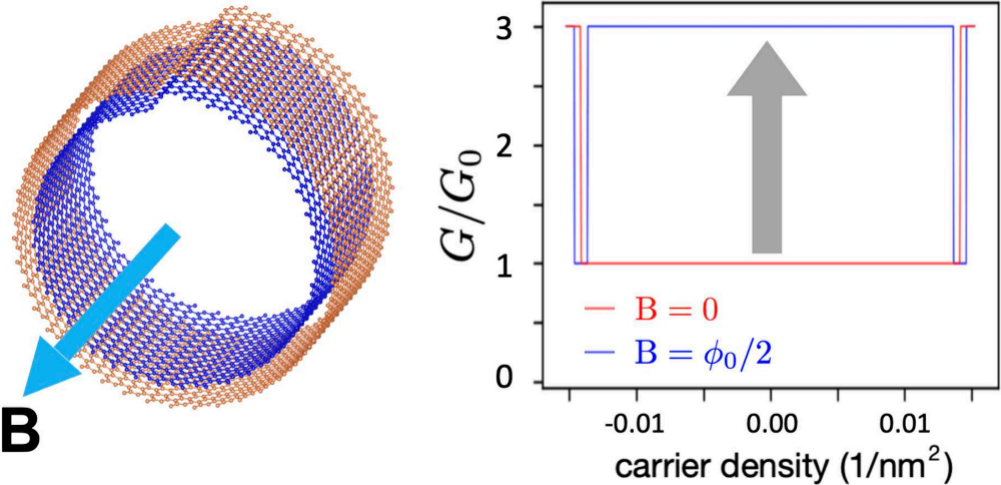

We theoretically demonstrate that carbon nanoscrolls, spirally wrapped graphene layers with open end points, can be characterized by a large positive magnetoconductance. We show that when a carbon nanoscroll is subject to an axial magnetic field of several Tesla, the ballistic conductance at low carrier densities of the nanoscroll increases by about 200%. Importantly, we find that this positive magnetoconductance is not only preserved in an imperfect nanoscroll (with disorder or mild interturn misalignment) but can even be enhanced in the presence of on-site disorder. We prove that the positive magnetoconductance comes about with the emergence of magnetic-field-induced zero-energy modes, specific to rolled-up geometries. Our results establish curved graphene systems as a new material platform displaying sizable magnetoresistive phenomena.

Magnetocondutance, the change of conductance in response to an externally applied magnetic field, appears in different magnetic and nonmagnetic materials alike and can have various physical origins. At very low temperatures, the presence of quantum interference effects, specifically weak (anti)localization, leads to a positive (negative) magnetoconductivity.^1^ Weak antilocalization has recently been observed, for instance, in topological insulators^2^ and is related to the strongly spin–orbit-coupled Dirac surface states of these materials. The competition between weak localization and antilocalization in InGaAs-based two-dimensional (2D) systems was analyzed through magnetoconductance.^3^ In the 2D electron gas formed at LaAlO_3_/SrTiO_3_ interfaces, a combination of spin–orbit coupling and scattering by finite-range impurities gives rise to a single particle mechanism of positive magnetoconductance in response to in-plane magnetic fields and at temperatures of up to the 20 K range.^4^ Furthermore, the negative longitudinal magnetoresistance of Weyl semimetals^5^ is connected to the chiral anomaly of Weyl Fermions. It can reach values of up to 40% and exhibits a strong angular dependence.^6,7^

In nanostructures, geometrical effects due to atomic structures,^8,9^ strain and defect engineering,^10^ or layer stacking^11^ can be the platform for magnetoresistive phenomena as well.^12^ For instance, the ballistic magnetoconductance calculated in a carbon nanotube reveals a steplike structure as a function of magnetic flux.^13^ In topological insulator (TI) nanowires, the π Berry phase due to the spin-momentum locking of the surface states leaves its hallmark on the electronic band structure and provides a gap in the energy spectrum.^14^ When threaded by a half magnetic flux quantum, the surface state gap effectively vanishes,^15^ thereby implying a positive magnetoconductance^16,17^ and Aharonov–Bohm oscillations. Additionally, magnetotransport has been theoretically studied in shaped TI nanowires, such as nanocones and dumbbells,^18,19^ where the surface electrons experience an out-of-plane component of the coaxial magnetic field. This variation in the cross-sectional area leads to unconventional magnetic transport properties. Furthermore, geometrical effects have also been shown to lead to dipolar distributions of Berry curvature^20−22^ and consequently to the observation of a nonlinear Hall effect in the presence of time-reversal symmetry.^20^

In this study, we focus on the magnetotransport properties of carbon nanoscrolls (CNSs) with a turn number of two or fewer.^23−26^ This compact nanoarchitecture can be synthesized by rolled-up technology and can be seen as radial superlattices due to their spiral cross section. This results in a very peculiar band structure and transport behavior different from conventional flat nanostructures.^27,28^ Both blue phosphorus^29^ and black phosphorus nanoscrolls^30^ are characterized by high carrier mobility. Importantly, there has been growing attention on aluminum- and lithium-based batteries that make use of carbon-based radial-superlattice cathodes.^31−35^

The main findings of our study are summarized in Figure 1. In a two-turn CNS with zigzag edges (Figure 1a), the ballistic conductance is tripled when the nanostructure is threaded by a half-integer magnetic flux quantum ϕ_0_/2 (Figure 1b). This translates into a positive magnetoconductance coefficient (PMC) that reaches 200%. Remarkably, at low carrier densities, the ballistic conductance of a CNS is only weakly affected by disorder (Figure 1c). This is in sharp contrast to a graphene zigzag ribbon that displays a zero-conductance dip.^36^

**Figure 1:**
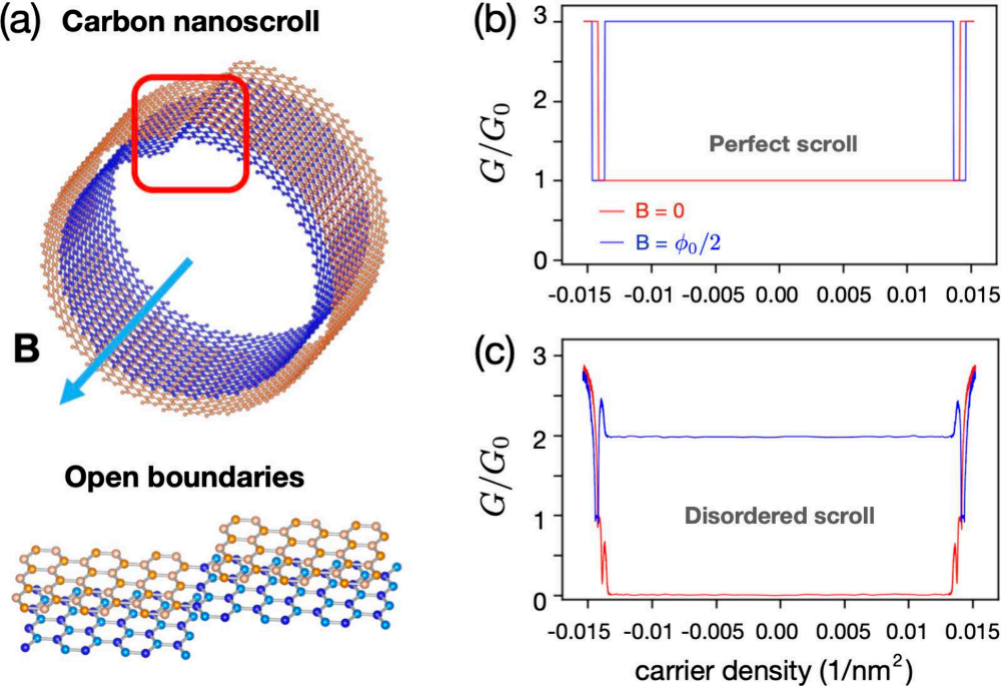
Conductances of two-turn CNSs with and without applied magnetic fields. (a) Way of applying magnetic fields and the boundary conditions of the interface. (b) Conductances for the case of without disorder. (c) Conductances for the case of with disorder. In parts b and c, the red line indicates the applied magnetic field *B* = 0 T, whereas the blue line is for *B* ≈ 10 T (10.3949 T in the numerical calculation).

In order to analyze ballistic transport in CNSs, we employ both a continuum **k**·*p* model^37^ and a tight-binding model, with which we perform numerical calculations using the Kwant package.^38^ In the following, we consider a two-turn CNS that can be mapped to bilayer graphene (Figure 1a) with mixed boundary conditions.^37^ The corresponding four-band continuum model takes into account the sublattice and layer degrees of freedom and can be written in the A1, B1, A2, and B2 basis.^39^ The resulting energy dispersion can be obtained from the relation , where *k*_±_ and *k*_*z*_ are the momenta in the tangential and axial directions of the CNS, respectively. In this equation, we introduce the velocity  with lattice constant *a* (see Section S1 in the Supporting Information).

We construct a tight-binding model for a CNS by rolling a zigzag graphene nanoribbon perpendicular to its edges, restricting it to AB-stacked (Bernal-stacked) structures. The model accounts for nearest-neighbor and interlayer hoppings. The unit cell consists of pairs of A–B carbon atoms from the nanoribbon, represented as {A_1_, B_1_; A_2_, B_2_; ...; A_*m*_, B_*m*_}, as shown in Figure 2a, where *m* is the number of pairs of A–B carbon atoms in the unit cell. Along the zigzag boundaries, the carbon atoms of different layers are also aligned according to the AB-stacking configuration. To form a two-turn CNS with an AB-stacked structure, an example with 7 pairs of A–B carbon atoms (*m* = 7) is presented in Figure 2b,c.^40,41^ In this structure, half of the atoms sit above the centers of the hexagons, while the others are directly above the atoms of the inner layer.

**Figure 2:**
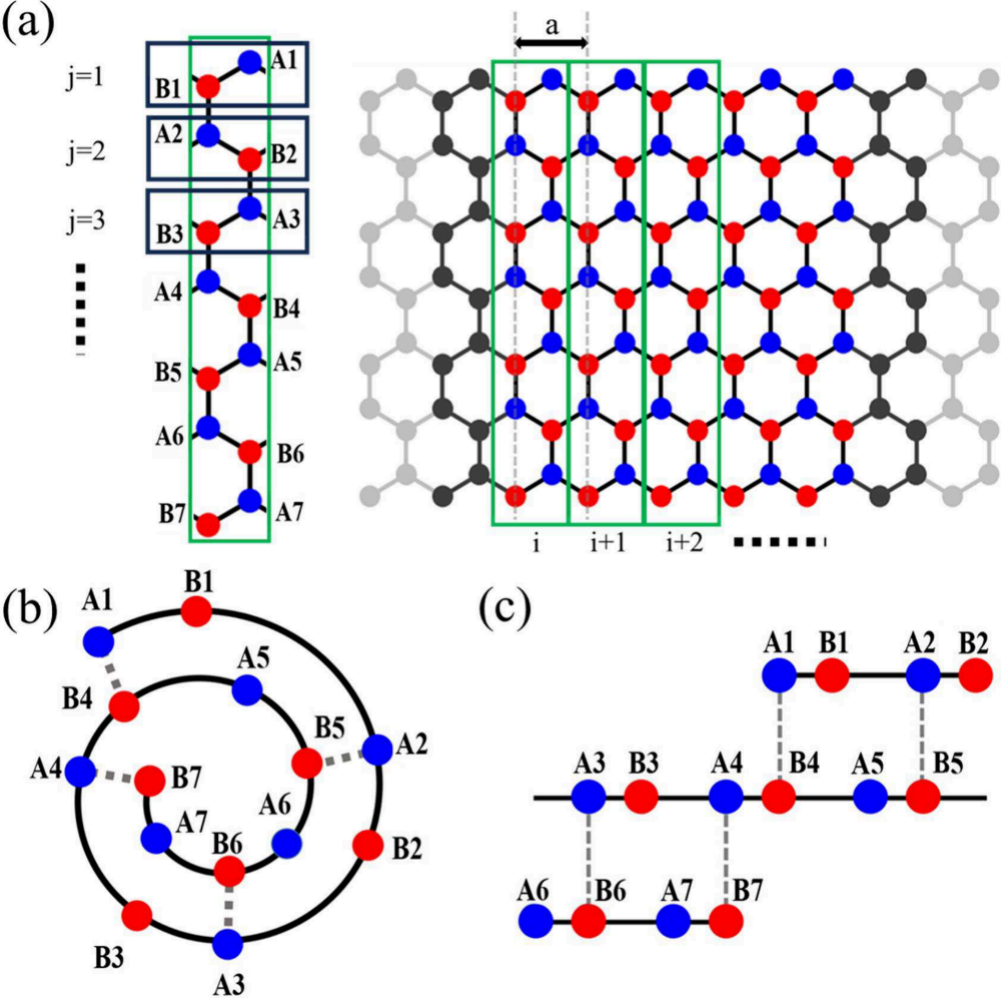
(a) Unit cell, denoted by a green rectangular, of the CNS defined along the axis perpendicular to the core axis and with open boundaries. The length of the unit cell is determined by the arc length of the nanoscroll. (b) Cross section of a CNS featuring two turns. (c) Flattened interlayer structure of the CNS showing an AB-stacking arrangement. The dashed lines in parts b and c denote the interlayer coupling γ_1_.

For modeling the two-turn CNS, we fix the intralayer coupling strength between A and B sites at γ_0_ = 3.16 eV and the interlayer coupling strength between site A2 (A site in the second turn) and site B1 (B site in the first turn) with γ_1_ = 0.381 eV, corresponding to an interlayer distance of 3.35 Å in the AB-stacked bilayer graphene.^39,42,43^ The lattice constant *a* is 2.4595 Å^44^ with a carbon–carbon bond length of 1.42 Å for graphene. For the system length scale, we set the total arc length to *X* = 100 nm, which contains 934 atoms, for both the two-turn nanoscroll and the Möbius tube in the Kwant simulation.^45^ This corresponds to a perimeter of *L* = 50 nm and a radius of 7.99 nm for a single turn. Furthermore, the length along the core axis is 300 nm.

We define the PMC as
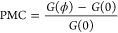
1where *G*(ϕ) indicates the conductance with magnetic flux ϕ. The two-terminal conductance in the ballistic regime is given by the Landauer formula^37,46−48^
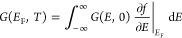
2where *f* is the Fermi–Dirac distribution function and *E*_F_ is the Fermi energy. The zero-temperature perfectly ballistic conductance of our one-dimensional (1D) nanostructure is proportional to the number of modes (*N*_s_) and given by *G*(*E*,0) = 2e^2^*N*_s_ /*h*. We neglect the mild spin–orbit coupling of graphene.

To account for the effect of disorder, we include a random on-site potential that is Gaussian-distributed^37,49^ (see Section S2 in the Supporting Information). We consider two characteristic disorder strengths of 0.1 and 0.5 eV, respectively, both comparable to the intralayer hopping amplitude. We examined the convergence of the averaged conductance and found that 200 configurations already achieved a small fluctuation of 5%. Therefore, we use 200 random disorder configurations for the results presented in this paper, unless otherwise stated. More details on disorder convergence tests and, in addition, the calculations for the localization length, proportional to the mean free path in a (quasi-)1D system,^50^ are provided in Section S2 of the Supporting Information.

To get a comprehensive understanding of the transport properties of a two-turn CNS threaded by a magnetic flux, we first study the two-terminal conductance of a monolayer graphene nanoribbon with zigzag edges and ribbon width equal to the total arc length of our two-turn nanoscroll (Figure 3a). Based on an order-of-magnitude estimation, we expect that the Zeeman effect and spin–orbit coupling have a negligible impact on the large PMC.^51,52^ Parts e and i of Figure 3 show that, in the low carrier density regime (electron or hole carrier density lower than 0.015 nm^–2^) and thus close to the charge neutrality point (Fermi energy |*E*| < 30 meV), the ballistic conductance is simply given by *G*_0_. Disorder leads to zero-conductance dips close to the charge neutrality point (see the red and blue lines in Figure 3e,i). Very similar features are encountered when considering either a bilayer graphene ribbon (Figure 3f,j), a two-turn CNS in the absence of externally applied fields (Figure 3g,k), or a double-walled carbon nanotube (see the Supporting Information).

**Figure 3:**
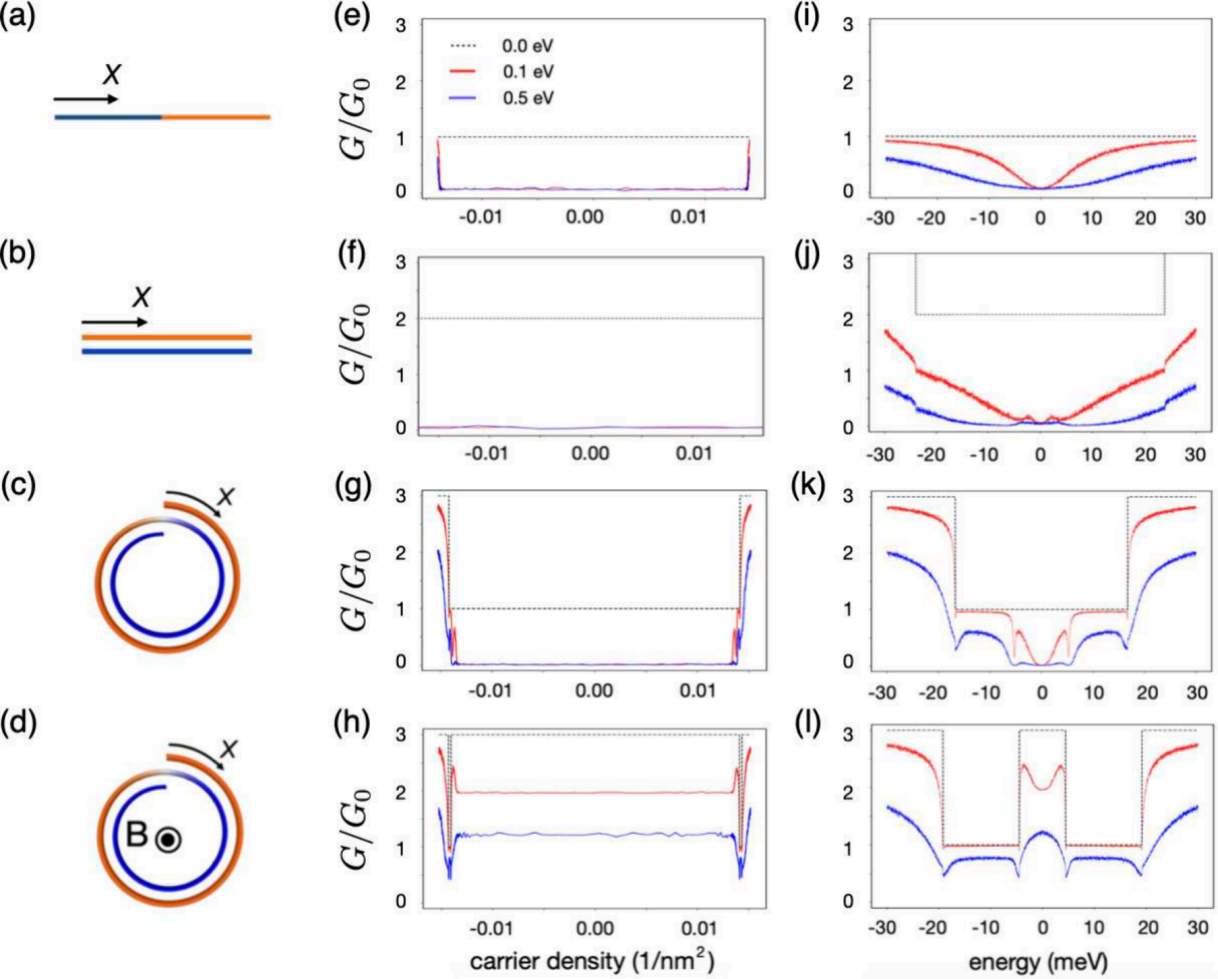
Conductances of (a) a monolayer ribbon, (b) a bilayer ribbon, (c) a two-turn CNS, and (d) a two-turn CNS with applied magnetic flux for different disorder strengths. (e) Conductances of a monolayer ribbon, i.e., γ_1_ = 0 eV. (f) Conductances of an AB-stacked bilayer ribbon with interlayer coupling strength γ_1_ = 0.381 eV. (g) Conductances of two-turn CNSs without applied magnetic fields. (h) Conductances of two-turn CNSs with applied magnetic fields *B*≈ 10 T (10.3949 T in the numerical calculation). As the counterparts of parts e–h, parts i–l show the conductances with the *x*-axis resenting energies. The gray dashed line denotes the conductance for the perfect lattice. The red and blue lines indicate the results of a system with applied disorder 0.1 and 0.5 eV, respectively.

For a CNS threaded by a half-integer magnetic flux quantum (Figure 3d), the situation is completely different. As shown in Figure 3h,l, the ballistic conductance at charge neutrality is tripled, compared to the results of the monolayer nanoribbon shown in Figure 3e,i. Furthermore, adding disorder does not lead to any zero-conductance dip even for a disorder strength of about 0.5 eV and thus larger than the interlayer hopping amplitude (see the blue line in Figure 3h,l). We thus find that a CNS is characterized by a PMC that reaches 200% near the charge neutrality point. Moreover, the localization length along the core axis of a two-turn CNS with a magnetic flux exceeds 1 μm, as detailed in Section S2 of the Supporting Information. This indicates that PMC can be realized in nanoscroll systems with length scales ranging from nanometers to micrometers.^23,26^

We note that the additional phase of the nanoscroll states, determined by the applied magnetic flux, is given by , where *B* is the magnetic field strength and *L* is the one-turn length of the nanoscroll. For our proposed magnetotransport to occur at ϕ = π/2 = π(*L*/2π)^2^*B*_c_, the required magnetic field strength *B*_c_ can be reduced by a factor of *N*^2^ times by increasing the nanoscroll’s arc length by a factor of *N*. For a two-turn nanoscroll with an arc length of 150 nm, for example, the required field strength, achieving the results shown in Figure 3h,l, can be reduced to approximately 4.6 T (see Section S3 in the Supporting Information). Additionally, the energy and conductance under various applied magnetic fields are presented in Section S4 of the Supporting Information.

The conductance tripling in a CNS threaded by a half-integer magnetic flux quantum can be understood by considering the electronic characteristics of CNSs. We start by considering a Möbius-like geometry in which the open end points of the CNSs are closed (Figure 4a). Close to the K (K′) valley, we observe the appearance of 2-fold degenerate zero-energy modes. This zero-energy mode disappears when opening the boundary conditions as in an actual CNS (Figure 4b). Instead, we observe the appearance of the characteristic zero-energy edge modes of zigzag-terminated graphene. With a half-integer magnetic flux quantum, the energy spectrum for a Möbius-like CNS does not qualitatively change; we only observe a shift in the axial momentum of the doubly degenerate zero-energy modes (Figure 4c). The case of a CNS with open boundary conditions threaded by a magnetic flux retains the zero-energy zigzag edge states found in the absence of magnetic fields. However, we concomitantly find the emergence of the zero-energy doublet with closed boundary conditions (Figure 4d). It is the appearance of these additional modes that leads to the tripling of the ballistic conductance in the vicinity of the charge neutrality point (the region of low carrier densities).

**Figure 4:**
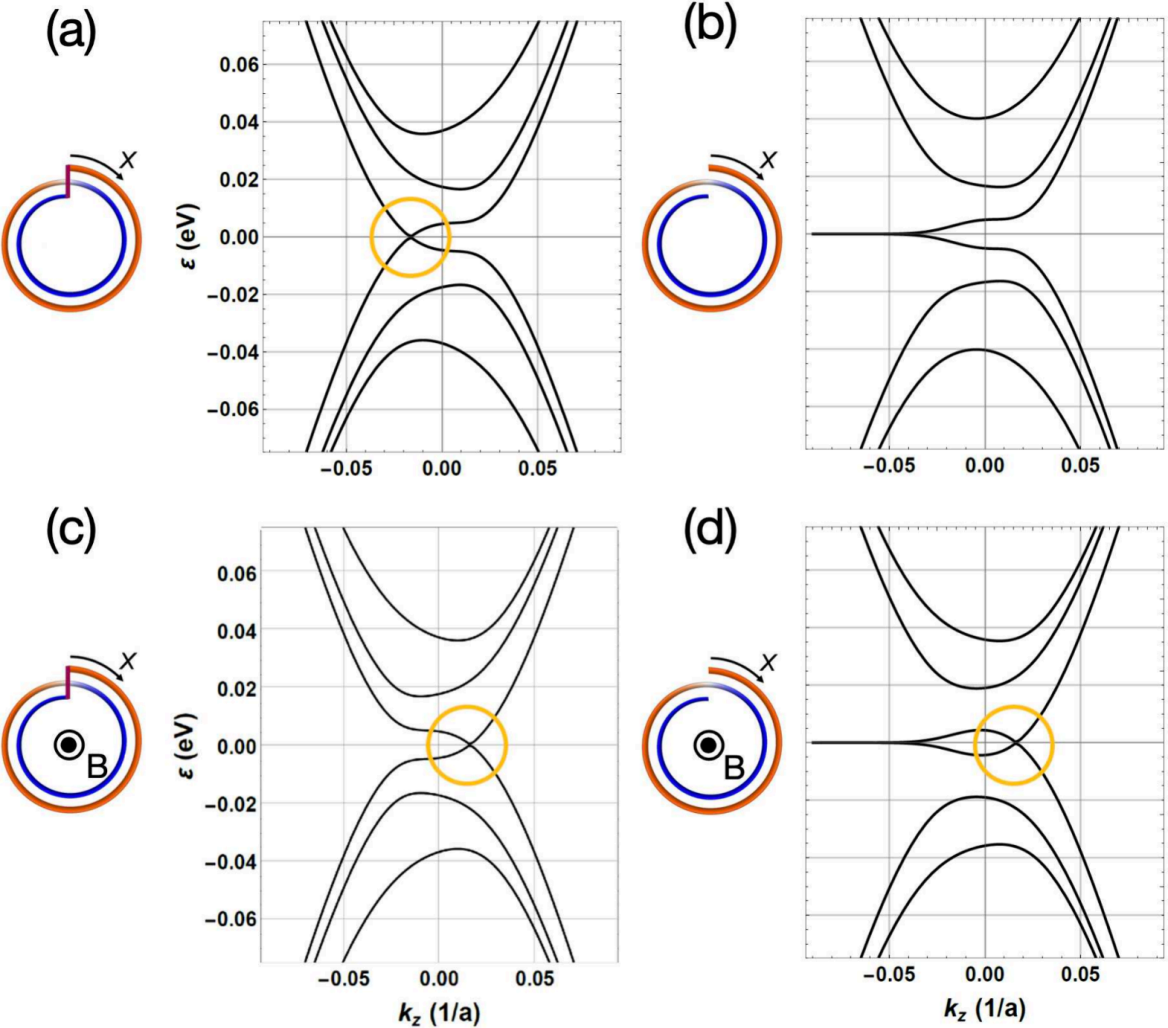
Energy bands of the two-turn CNSs and Möbius tube for the K point: (a) Möbius tube and (b) two-turn CNSs without applied magnetic fields; (c) Möbius tube and (d) two-turn CNSs with applied magnetic field *B*≈ 10 T (10.3949 T in the numerical calculation).

To further demonstrate that the doubly degenerate states at zero energy in the CNS with a magnetic flux are inherited from the nontrivial interfacial states in the Möbius-like CNS, we estimated the charge density distributions of the zero-energy states in the Möbius-like CNS, the Möbius-like CNS with an applied magnetic flux, and the CNS with the same magnetic flux. The results, shown in Figure 5, confirm this connection. We emphasize that pioneering studies^53−56^ have shown that the AB–BA interface in bilayer graphene induces a topological feature in *k* space, resulting in 1D interfacial topological valley states. Our findings in Figures 4 and 5 demonstrate that the nontrivial interfacial state is sustained not only in a Möbius tube with the same interface but also in a CNS under an applied magnetic field.

**Figure 5:**
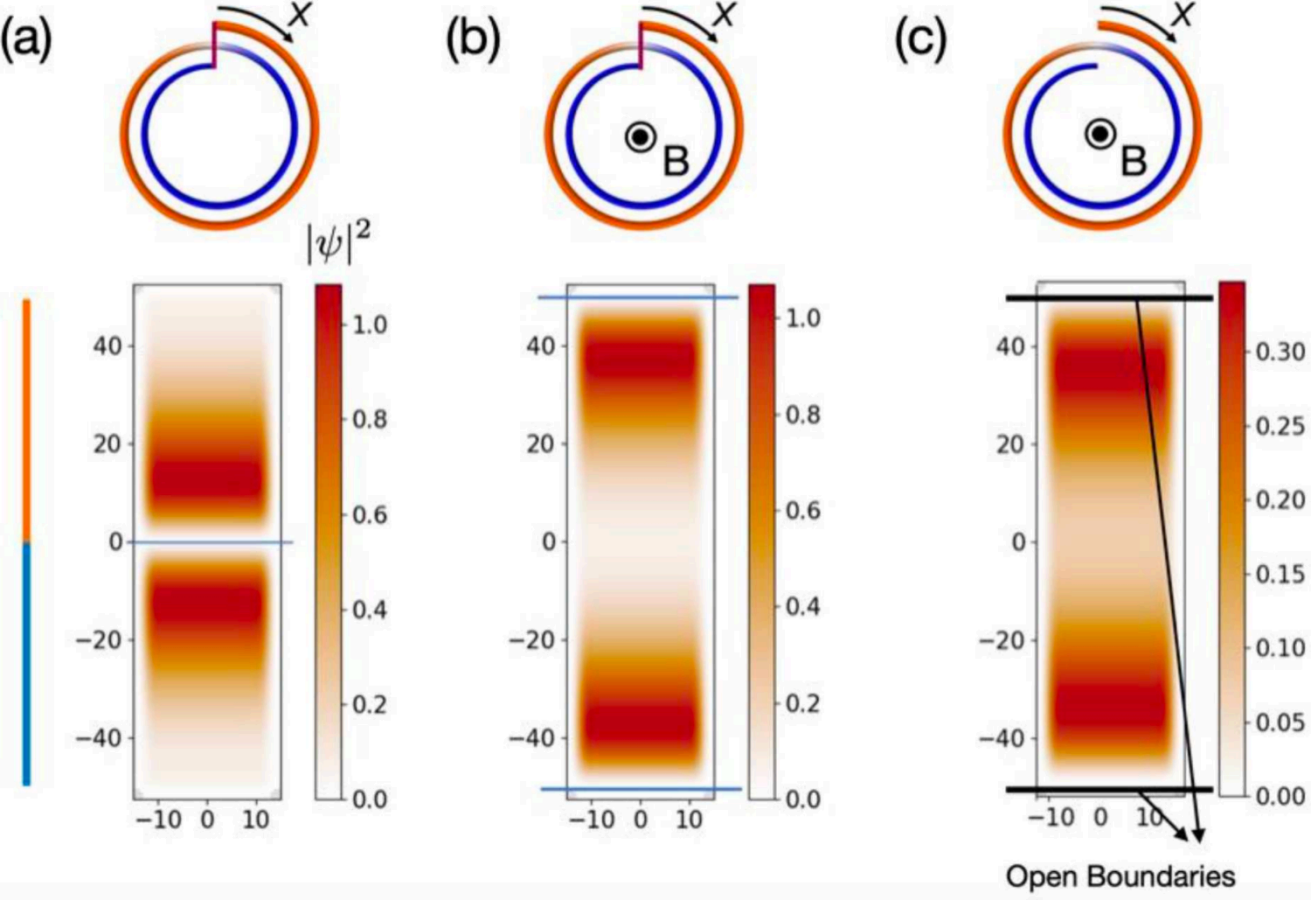
Distributions of charge densities of the doubly degenerate zero-energy states in (a) a Möbius tube, (b) a Möbius tube with an applied magnetic flux, and (c) a CNS with an applied magnetic field. The results are obtained by calculating tight-binding models of the Möbius tube and CNS.

In summary, we theoretically demonstrated that radial superlattices, especially in AB- and BA-stacked domain walls featuring two-turn CNSs with magnetic flux, display a giant PMC. We found that the PMC of a two-turn CNS is giant and up to more than 2 times that of the ordinary graphene nanoribbon. With simulations of disordered systems, we found that its conductance is less prone to disorder and PMC even increases, in contrast to the disordered TI nanowire in which PMC decreases remarkably.^14,15^

To interpret this novel result, we developed a model of the Möbius tube with an AB–BA bilayer interface and compared its band structures and quantum states with and without magnetic flux. The proposed PMC stems from nontrivial interfacial magnetic states, enabling it to persist not only under on-site disorder but also in systems with moderate lattice misalignment (see Section S5 in the Supporting Information) or an imperfect turn number in the nanoscroll (see Section S6 in the Supporting Information). It is expected that the insights and effects that we unveiled in our work will be observed in the experimental field.

## Supporting Information Available

Details of the continuum model, disordered simulation, localization length, conductance at different magnetic fields, and magnetoconductance for misaligned and 1.8-turn nanoscrolls (PDF)
