# Supplementary material for: Large Positive Magnetoconductance in Carbon Nanoscrolls
Source: Nano Lett. 2025 Mar 28;25(14):5550–6. doi: 10.1021/acs.nanolett.4c03694 (PMC11987012; doi:10.1021/acs.nanolett.4c03694)
Supplement: Supplementary file 1 — nl4c03694_si_001.pdf [file nl4c03694_si_001.pdf]

Large positive magnetoconductance in carbon nanoscrolls: Supporting Information

Yu-Jie Zhong,[†,‡] Jia-Cheng Li ,[¶,‡] Xuan-Fu Huang,[†,‡] Ying-Je Lee,[†,‡] Ting-Zhen Chen,[†,‡] Jia-Ren Zhang,[†,‡] Angus Huang,[§] Hsiu-Chuan Hsu,[*,‖] Carmine Ortix,[⊥] and Ching-Hao Chang[*,†,‡,¶]

†*Department of Physics, National Cheng Kung University, Tainan 70101, Taiwan*

‡*Center for Quantum Frontiers of Research and Technology (QFort), National Cheng Kung University, Tainan 70101, Taiwan*

¶*Program on Key Materials, Academy of Innovative Semiconductor and Sustainable Manufacturing, National Cheng Kung University, Tainan 70101, Taiwan*

§*Department of Physics, National Tsing Hua University, Hsinchu 30013, Taiwan*

‖*Graduate Institute of Applied Physics, National Chengchi University, Taipei 11605, Taiwan*

⊥*Dipartimento di Fisica "E. R. Caianiello", Università di Salerno I-84084 Fisciano (Salerno), Italy*

E-mail: hcjhsu@nccu.edu.tw; cutygo@phys.ncku.edu.tw

S1. Continuum model

To concretely prove the appearance of such unconventional remarkable conductance and disorder resistant transport, we model[1] a two-turn CNS and Möbius tube with the low-energy $\mathbf{k} \cdot \mathbf{p}$ of bilayer graphene along the arclength (X axis) of the nanostrucure as the

tunneling occurring between the two layers in the special boundary conditions as shown in **Fig. 1(a) and (b)**.

Specifically the effective four band model for the $2p_z$ orbitals is utilized on the four atomic sites $A1, B1, A2, B2$ of the unit cell. We have the effective four band model written as follows:[2]

$$H_b = \begin{pmatrix} 0 & v\pi^\dagger & 0 & 0 \\ v\pi & 0 & \gamma_1 & 0 \\ 0 & \gamma_1 & 0 & v\pi^\dagger \\ 0 & 0 & v\pi & 0 \end{pmatrix}, \tag{1}$$

where the momentum operators are given by $\pi = -i\hbar(k + i\xi k_z)$ and $\pi^\dagger = i\hbar(k - i\xi k_z)$ with k_z along the CNS axis and k along the arclength direction. ξ is the valley index distinguishing between the two inequivalent K points in the Brillouin zone. The Fermi velocity is read as $v = \sqrt{3}a\gamma_0/2\hbar$ with γ_0 denoting the intralayer hopping amplitude between sites A1 and B1 (or A2 and B2). Finally, we explicitly consider the the dimer sites A2 and B1 interlayer coupling γ_1.

The energy ε for Eq. (1) is obtained by $\hbar v k_\pm = \sqrt{\varepsilon^2 \pm \gamma_1\varepsilon - \hbar^2 v^2 k_z^2}$ with $\pm$ as the valence and conduction bands. Moreover, the left-moving L and right-moving R wave functions are read as

$$\Phi_\pm^{R(L)} = N_\pm \begin{pmatrix} \mp i\hbar v\left[(-)k_\pm - i\xi k_z\right] \\ \mp\varepsilon \\ \varepsilon \\ -i\hbar v\left[(-)k_\pm + i\xi k_z\right] \end{pmatrix} e^{(-)ik_\pm X + ik_z z}, \tag{2}$$

where $N_\pm$ is a normalization constant.

In the continuum model, the electronic band structure of the two-turn CNS can be given

by the boundary conditions:

$$\begin{aligned}
\Psi_{A2}(2\pi R) &= \Psi_{A1}(0) \\
\Psi_{B2}(2\pi R) &= \Psi_{B1}(0) \\
\Psi_{A1}(2\pi R) &= 0 \\
\Psi_{B2}(0) &= 0,
\end{aligned} \tag{3}$$

with R being the radius of the CNSs and $L = 2\pi R$ being the total arclength of each layer. Similarly, the boundary conditions for the Möbius tube are given by, if the open endpoints in CNSs rejoin the inference (closed endpoints):

$$\begin{aligned}
\Psi_{A2}(2\pi R) &= \Psi_{A1}(0) \\
\Psi_{B2}(2\pi R) &= \Psi_{B1}(0) \\
\Psi_{A2}(0) &= \Psi_{A1}(2\pi R) \\
\Psi_{B2}(0) &= \Psi_{B1}(2\pi R).
\end{aligned} \tag{4}$$

To fit the boundary conditions Eq. (3) and Eq. (4), we write a hybrid wave function Ψ as generic wave function, which is a combination of the left-moving L and right-moving R wave functions in the conduction and valence bands $\Phi_{\pm}^{R(L)}$. In other words, the generic wave function is written as $\Psi = a\,\Phi_{+}^{L} + b\,\Phi_{-}^{L} + c\,\Phi_{+}^{R} + d\,\Phi_{-}^{R}$, where a, b, c, and d are coefficients that we fix by applying the boundary conditions.[1]

We show the properties of charge density of two-turn CNS as follows. **Fig. 1** gives the profiles of charge density of CNSs with and without applied magnetic fields **Fig. 1(a)** and **Fig. 1(b)** are for the case without applied magnetic field, and **Fig. 1(c)** We can discover that in the case of two-turn CNS without applied magnetic field, the charge density is more focused on the open endpoints and the interface, as **Fig. 1(a) and (b)**. Yet, while the magnetic field is applied, the value of charge density is slightly flattened at the

open endpoints and the interface; on the contrary, we can find that charge density increases overall on the rest part of the arclength of CNSs, as **Fig. 1(c) and (d)**.

We note that the charge density is highly focused on the interface and the open endpoints in the CNS while the applied magnetic field approaches zero, as **Fig. 1(a) and (b)**. Yet, in **Fig. 1(c) and (d)**: CNSs with the applied magnetic field, the decreasing of charge density on the interfaces and two open endpoints, yet the increase on the rest part of archlength of CNS indicates one thing: the probability in average of scattering for the CNS increases when the magnetic field is applied. This also drops us the hint to interpret why CNSs with applied magnetic fields possess highly disorder tolerant conductance.

Additionally, we present a charge density profile for four sites of two-turn CNSs under the applied magnetic field, as shown in **Fig. 2**. We concentrate on the gapless energy crossing point and illustrate the significant aggregation of charge density.

S2. Simulation results for disordered CNSs

We provide the conductance that is calculated by the tight-binding calculation with the open-source python package Kwant,[3] and consider point defect disorder applied to CNSs. We simulate the transport with the disorder and model each of the lattices in possession of randomly onsite energy with a Gaussian distribution :[1,4]

$$p(x) = \frac{1}{\sqrt{2\pi\sigma^2}} e^{-\frac{(x-\mu)^2}{2\sigma^2}}, \tag{5}$$

where the parameter μ is the expectation and σ^2 the variance of the distribution $p(x)$. In Eq. (5), the intensity of impurity σ as disorder is considered as

$$\begin{aligned} \sigma(y) &\propto e^{\frac{y}{X}}; \qquad y < \frac{X}{2} \\ \sigma(y) &\propto e^{\frac{(L-y)}{X}}; \qquad y > \frac{X}{2}, \end{aligned} \tag{6}$$

where y denotes as the position of atom that the onsite disorder applied in the real space. X is the arclength of CNS.

S2.1. The convergence tests with more disorder configurations

To show the testing convergency for higher disorder configurations, we have increased the disorder configurations from 10 to 100 and 200. Additionally, we increase an order of magnitude for the calculated data points, from 200 to 4000, in the same energy range to check the convergence of the simulation. The results are shown in **Fig. 3**.

The panel (a) is our previous results for magnetoconductance of the two-turn nanoscroll with magnetic flux. We first keep the disorder configurations at 10 and increase the solved data point from 200 to 4000 to get an averaged numerical result. As shown in panel (b), the fluctuation of data points remains wide (10%-20% fluctuation amplitude). To reduce the fluctuation in panel (b), we keep the solved data points at 4000 and increase the disorder configurations from 10, 100 to 200, shown in panels (b), (c), and (d), respectively. By comparing the results in panels (c) and (d), where the fluctuation amplitude is less than 5%, we justify that the disorder average converges to within 5% when the number of disordered samples is larger than 100.

In comparison to graphene nanoribbon, the carbon nanoscroll with doubly connected geometry has a faster convergence as a function of the number of configurations. This nature is identified in **Fig. 4** comparing the results of a graphene nanoribbon (panel (a)) and a nanoscroll (panel (b)) with the same arclength. For the same Fermi energy at 10 meV, the width of conductance fluctuation driven by random disorder in the range ± 0.1 eV reduces three times when the graphene rolls up into the carbon nanoscroll (see gray solid lines in the panels (a) and (b)).

S2.2. Calculation of localization length

To approximately obtain the localization length, a length scale proportional to the mean free path in 1D system, we fix the on-site disorder strength at 0.1 eV and alter the axial length (the length along core axis) of nanoscroll from 5, 6, 7 ... to 300 nm. Additionally, we compute the average conductance over 100 disorder configurations at Fermi level $E = 0.0001$ eV.

To make a comparison between nanoscroll magnetic state and graphene edge states, we also do the same calculation of conductance for a graphene zigzag ribbon with the same width and length. The results are shown in **Fig. 5**. As shown in panels (a) and (b), the conductance in all nanoarchitectures decays with the extension of axial length. In nanoscroll with magnetic flux, however, the conductance decays much slower than the nanoscroll without magnetic flux and graphene nanoribbon. The localization length λ can be directly obtained in panel (b) by fitting the numerical results for $Y = 100$ to 300 nm. The mean free paths are approximately 1600 nm, 96 nm, and 139 nm for nanoscroll with magnetic flux, nanoscroll, and graphene nanoribbon, respectively.

The localization length for the nanoscroll with magnetic flux is at least one order of magnitude larger than other systems. The localization lengths in nanoscroll and nanoribbon states are in the same order around a hundred nm. It can be expected that the edge states in graphene nanoribbon are sensitive to point defects that may destroy boundaries.

The calculations further reveal that the ground states associated with band crossing in a nanoscroll with magnetic flux are much more robust against disorder compared to the zigzag edge states in graphene.

S3.1. The conductance of two-turn (double-walled) carbon nanotubes

Regarding the origin of the +200% magnetoconductance and for performing a comparison by calculating the conductance of a carbon nanotube, we have calculated the conductance of a two-turn (double-walled) carbon nanotube. To facilitate comparison with the two-turn nanoscroll in our work, we simulate the nanotube with the same number of atoms and the same AB-stacked nanoarchitecture. The only difference between the two systems lies in the boundary conditions.

As shown in **Fig. 6(a)**, a band crossing appears at zero energy $E = 0$ in each valley in the nanotube. The band crossing splits when a magnetic field is applied along the core axis to drive a phase π in the system **Fig. 6(b)**. As a result, the applied magnetic field kills the low-energy conductance, providing "negative" magnetoconductance. Similar magneto-transport effects are reported in pioneer works that study monolayer (single-walled) carbon nanotubes.[5,6]

Moreover, the difference in magnetoconductance between the nanoscroll and the nanotube is not only in its sign – the conductance in carbon nanotube against the material disorder is not as robust as the nanoscroll. The conductance sharply drops to zero for the nanotube with onsite disorder (see blue line in panel (c)).

We have confirmed that the boundary conditions influence the positive magnetoconductance of +200% observed in nanoscrolls. Additionally, the positive magnetoconductance effect in nanoscrolls is more robust against disorder compared to nanotubes. This is because the degenerate magnetic ground states are spatially separated and localized on different types of sites (as shown in **Fig. 2**.) This separation helps reduce backscattering that is typically caused by disorder.

S3.2. Arc-length 150 nm CNSs

We provide the energy bands and the conductance with applied magnetic fields for the arclength 150 nm two-turn CNSs, as **Fig. 7** and **Fig. 8**. We observed the emergence of gapless crossing of energy bands at a magnetic field of 4.6 Tesla, equivalent to a half quantum flux.

S4. Conductance evolution with the applied magnetic field

We explore the behavior of the conductance of the perfect nanoscroll vs magnetic field, as shown in **Fig. 9**. This figure illustrates how conductance, as a function of energy, varies with the magnetic-field strength. The conductance of the carbon nanoscroll at low energy increases to three times its initial value, $3G_0$, when an approximately magnetic field ($B =$ 10.022 Tesla) is applied, corresponding to the induced energy crossing. We can further expect the evolution of the conductance response to the applied magnetic fields exhibits a periodic oscillation, as the nature of the Aharonov–Bohm effect in nanoscroll.[1] Consequently, the proposed positive magnetoconductance effect can appear at $(2n + 1)10.022$ Tesla, where n is an integer.

S5. Magnetoconductance for small interlayer misalignments of CNSs

To understand the impact of tiny interlayer misalignments on the predicted magnetoconductance that is driven by the magnetic ground states at the band crossings, we first examine the charge density distributions shown in **Fig. 2**. It is expected that a slight misalignment perturbatively modifies the interlayer hopping amplitude γ_1 which is the hopping amplitude

between the A2 site (the A site in the second layer) and the B1 site (the B site in the first layer). Notably, the charge density distributions of the A2 and B1 states are particularly low in the magnetic ground states, as illustrated in **Fig. 2**.

Therefore, we investigate the stability of the positive magnetoconductance in the presence of interlayer misalignment by simulating tiny and also remarkable disordered systems with random interlayer coupling strength γ_1. It is found that the random γ_1 only reduces magnetoconductance slightly near $E = 0$ (at ultralow carrier density), as is illustrated in **Fig. 10** (**Fig. 11**).

Moreover, the overlap probability between decaying tails of magnetic ground states is further reduced when the arclength of nanoscroll increases. As a result, the impact of interlayer misalignment in magnetoconductance becomes even weaker in a larger nanoscroll. This is confirmed in our new calculations provided in **Fig. 12** – the modulation of magnetoconductance driven by tiny misalignment becomes weaker in the nanoscroll with longer arclength.

Our new results in **Fig. 2** and **Fig. 10** to **Fig. 12** confirms that our predicted magnetotransport effect is robust against tiny misalignment.

S6. Comparison of 1.8-turn and two-turn carbon nanoscroll

To study the magnetotransport of a two-turn nanoscroll with zigzag boundaries, we reference pioneering works on graphene nanoarchitectures with unique boundary conditions and nontrivial interlayer structures, which inspired our approach. It has been noted that the domain walls of AB-BA stacked bilayer graphene in a bilayer ribbon can create topologically protected interfacial states,[7] which gives rise to one-dimensional valley-polarized conducting channels that are robust against material disorder.[8,9]

Inspired by the pioneer works, we expect that nontrivial quantum states can also be

hosted in a two-turn nanoscroll with zigzag edges for this system sharing the similar interfacial nanoarchitecture as the AB-BA stacked bilayer (see **Fig. 13(a)**). Moreover, the applied magnetic flux plays a role as a switch – we can turn on or off the nontrivial interfacial states in the nanoscroll by providing the phase in the states.

We first did the new calculation to confirm that the band structures at low energy are qualitatively the same as the two-turn nanoscroll for a nanoscroll with a turn number roughly less than two **Figs. S13(a) and (b)**. For a nanoscroll with a turn number larger than two; however, its band structure and respected transport behavior are qualitatively different from that of the two-turn nanoscroll with zigzag opened boundaries, as shown in our new calculations in **Fig. 14**. This is because the unique domain wall between AB and BA stacked bilayers cannot be established in a nanoscroll either with the number of turns being more than two.

For the nanoscroll with different chirality (armchair open boundary conditions), we can expect the system to have very different magnetic states and properties due to the lack of the unique domain wall between AB and BA stacked bilayers. We find that the armchair edge of the graphene ribbon does not create the ideal conditions for forming a perfectly connected interface, which possesses the perfectly AB-BA stacked domain wall interface and is crucial for the boundary conditions of a carbon nanoscroll. In contrast, the zigzag edge can successfully form a perfectly connected interface as a domain wall.

Moreover, we have estimated the magnetoconductance of a nanoscroll with the same number of atoms along the arc but only having 1.8 turns - a 20% fraction of the middle "layer" of the scroll not to have any interlayer coupling. Compared to the nanoscroll with two turns, the value and the energy range of magnetoconductance in the nanoscroll with 1.8 turns are kept and mildly extended, respectively, as shown in **Fig. 15**. This is due to the fact that the opened boundary conditions remain the same for the magnetic ground states induced in the 1.8 turns nanoscroll with magnetic flux. The region of the monolayer in the middle for a 1.8-turn nanoscroll only extends the decaying tails of magnetic ground states

(see following **Fig. 2**).

We show our prediction of positive magnetoconductance is not limited to two-turn nanoscroll with $X = 100$ nm. Although our prediction is based on the two-turn nanoscroll with zigzag open boundary conditions, we have confirmed that the proposed magnetotransport effect can occur in a nanoscroll with fewer turns (close to 2 turns, i.e., $\lesssim$2 turns) or with a different arclength (50 nm or 150 nm arclength).

References

(1) Zhong, Y.-J.; Huang, A.; Liu, H.; Huang, X.-F.; Jeng, H.-T.; You, J.-S.; Ortix, C.; Chang, C.-H. Magnetoconductance modulations due to interlayer tunneling in radial superlattices. *Nanoscale Horiz.* **2022**, *7*, 168–173.

(2) McCann, E.; Koshino, M. The electronic properties of bilayer graphene. *Reports on Progress in Physics* **2013**, *76*, 056503.

(3) Groth, C. W.; Wimmer, M.; Akhmerov, A. R.; Waintal, X. Kwant: a software package for quantum transport. *New J. Phys.* **2014**, *16*, 063065.

(4) Bardarson, J. H.; Brouwer, P. W.; Moore, J. E. Aharonov-Bohm Oscillations in Disordered Topological Insulator Nanowires. *Phys. Rev. Lett.* **2010**, *105*, 156803.

(5) Ajiki, H.; Ando, T. Aharonov-Bohm effect in carbon nanotubes. *Physica B* **1994**, *201*, 349–352.

(6) Wang, X.; Gao, W.; Li, X.; Zhang, Q.; Nanot, S.; Hároz, E. H.; Kono, J.; Rice, W. D. Magnetotransport in type-enriched single-wall carbon nanotube networks. *Phys. Rev. Mater.* **2018**, *2*, 116001.

(7) Zhang, F.; MacDonald, A. H.; Mele, E. J. Valley Chern numbers and boundary modes in gapped bilayer graphene. *Proc. Natl. Acad. Sci. U.S.A.* **2013**, *110*, 10546–10551.

(8) Ju, L.; Shi, Z.; Nair, N.; Lv, Y.; Jin, C.; Velasco, J., Jr.; Ojeda-Aristizabal, C.; Bechtel, H. A.; Martin, M. C.; Zettl, A.; Analytis, J.; Wang, F. Topological valley transport at bilayer graphene domain walls. *Nature* **2015**, *520*, 650–655.

(9) Geisenhof, F. R.; Winterer, F.; Seiler, A. M.; Lenz, J.; Martin, I.; Weitz, R. T. Interplay between topological valley and quantum Hall edge transport. *Nat. Commun.* **2022**, *13*, 1–7.

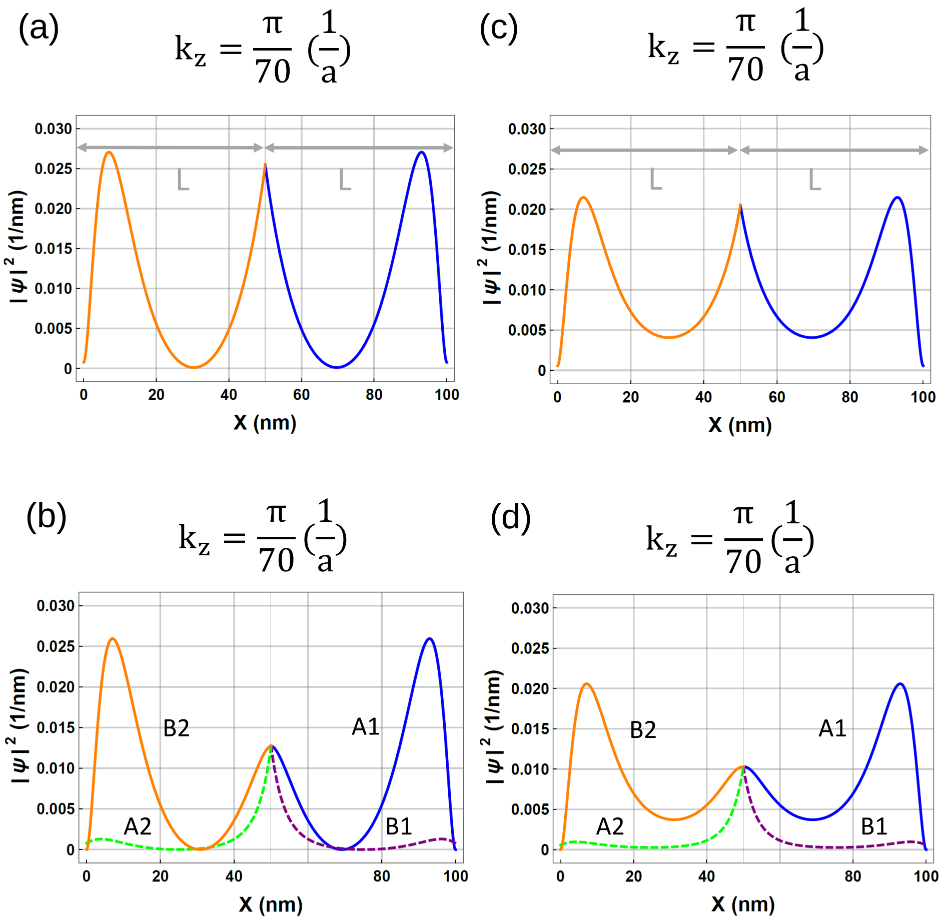

Figure 1: The charge density for the ground state of two-turn CNSs without and with applied magnetic fields. X is the arc length, and the interlayer coupling $\gamma_1 = 0.381$ eV. The left panel (a) and (b) are for without applied magnetic field cases, whereas the right panel (c) and (d) are with applied magnetic fields $B \approx 10$ Tesla (10.3949 Tesla in the numerical calculation). In (a) and (c), the orange line indicates the outer layer and blue line the inner layer of carbon CNSs. (b) and (d) are profiles of charge densities for the four sites, A1, B1, A2 and B2; the blue line indicates the A1 site, the purple dashed the B1, the green dashed the A2, and the orange the B2.

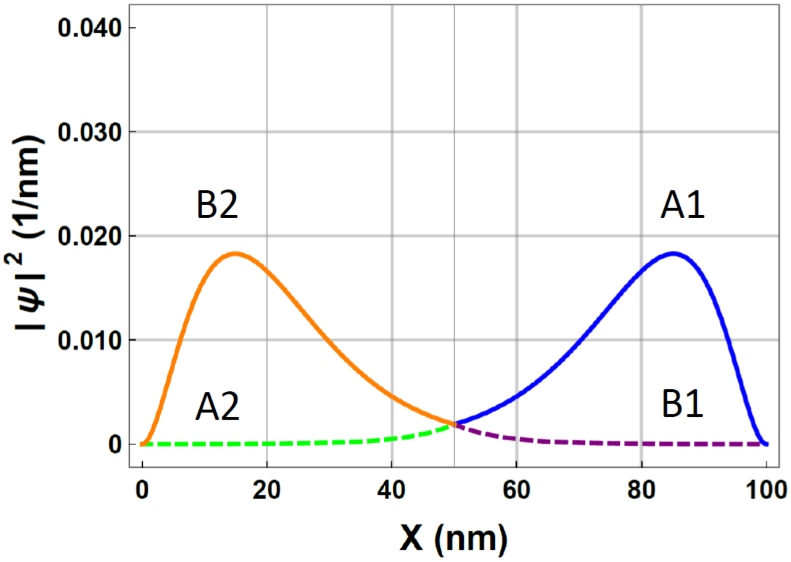

Figure 2: The charge density distributions at the gapless energy band crossing point $k_z = \frac{\pi}{191}$ (a^{-1}) along the arc for sites A1, A2, B1, and B2 in two-turn carbon nanoscrolls with an applied magnetic field are presented.

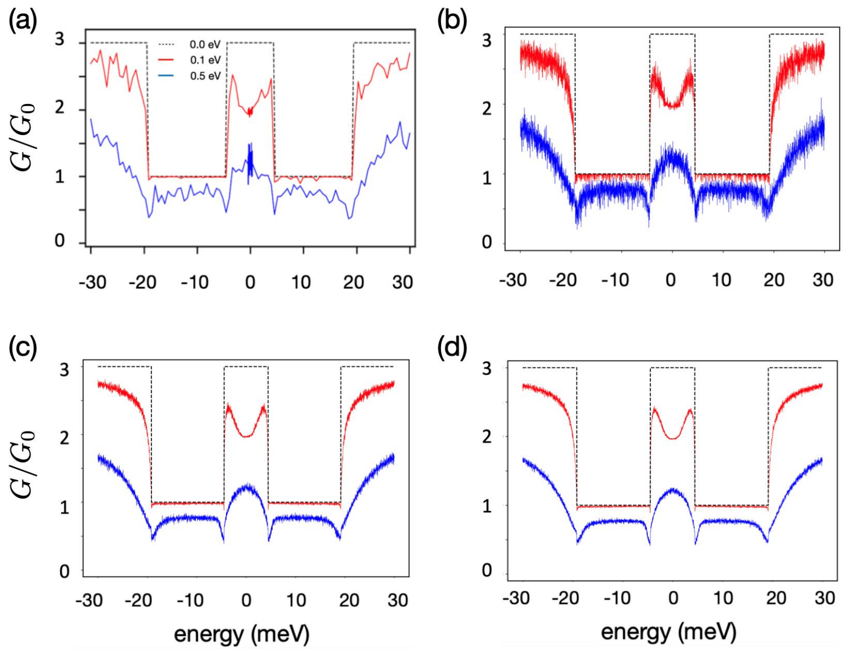

Figure 3: The disorder averaged magnetoconductance of the two-turn nanoscroll with magnetic flux. The dashed line is the conductance in the clean limit. The red (blue) line is the conductance in disordered systems with disordered strength of 0.1 (0.5) eV. (a) The number of disorder configuration is 10 and the number of data point is 200. In (b), (c) and (d), the number of data point is fixed at 4000 and the number of disorder configuration is 10, 100 and 200, respectively.

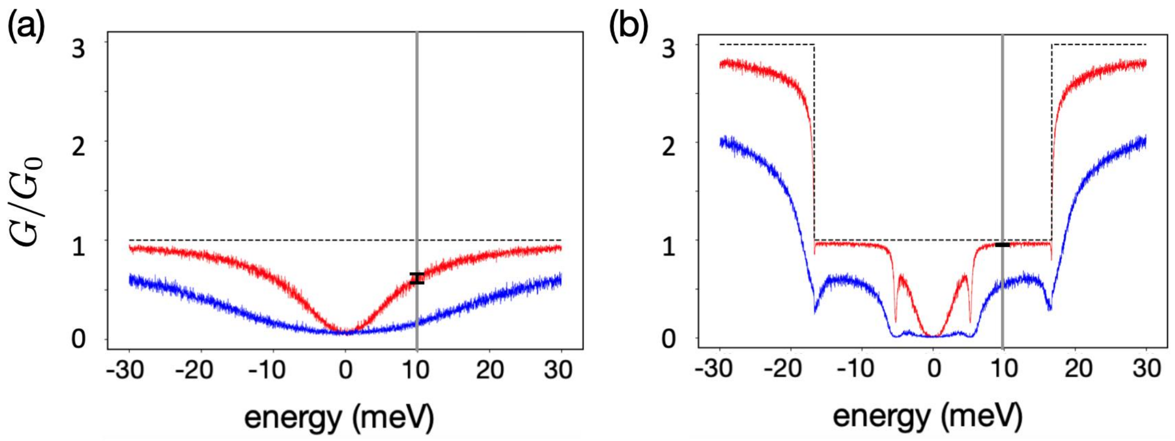

Figure 4: The conductance as a function of Fermi energy for graphene nanoribbon (a) and nanoscroll (b), averaged over 200 configrations. The dashed line is the conductance in the clean limit. The red (blue) line is the conductance in disordered systems with disordered strength of 0.1 (0.5) eV. The gray vertical lines pin points the Fermi energy at 10 meV.

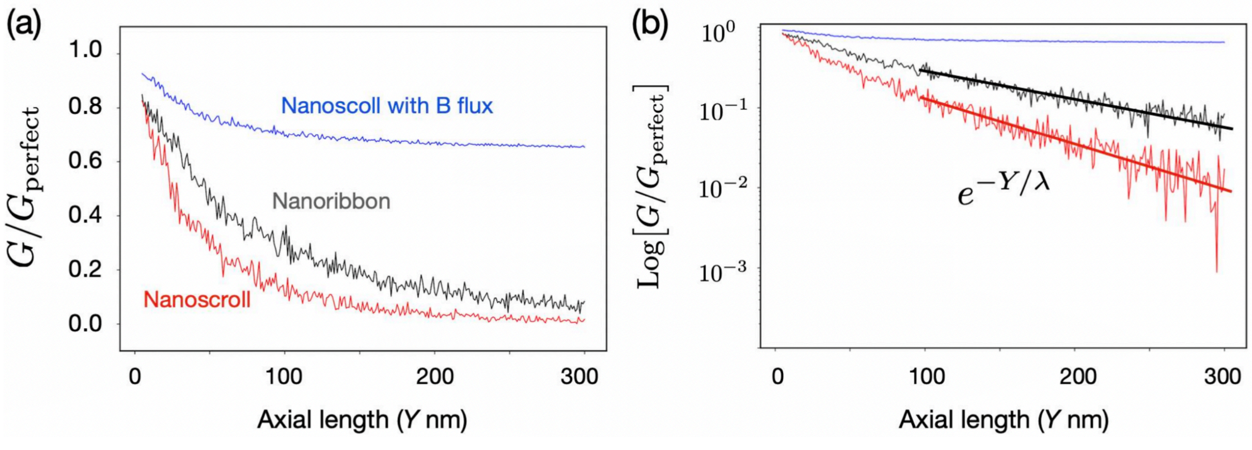

Figure 5: The conductance (a) and the log of the conductance (b) as a function with axial length for nanoscroll with magnetic flux (blue), nanoscoll (red) and nanoribbon (black). In (b), the fitted straight lines are plotted on top of the numerical data.

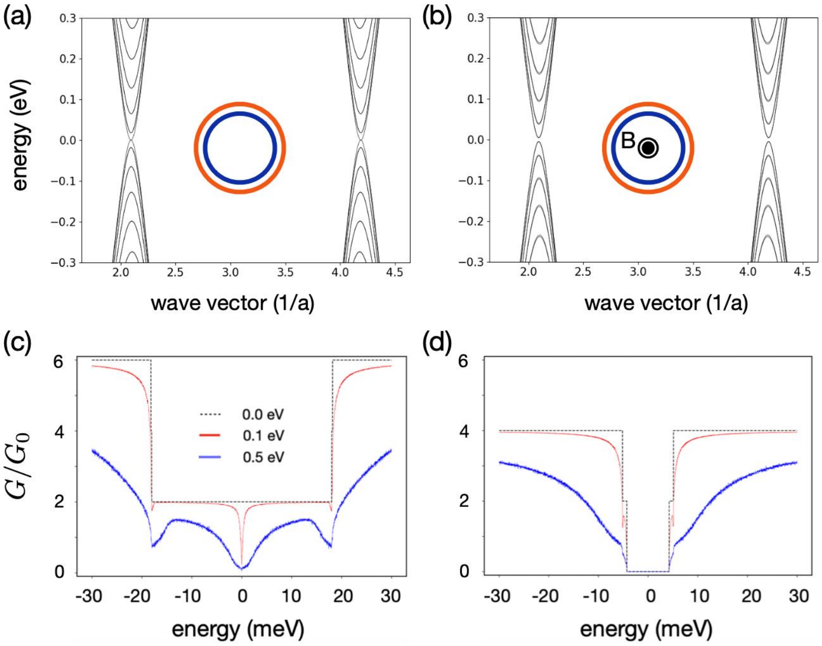

Figure 6: The band structure of a bilayer carbon nanotube (a) without and (b) with applied magnetic field along its core axis. Panel (c) and (d) are the conductance of the system in (a) and (b) simulated with the disorder, respectively. As defined in our manuscript, $G_0 = 2^2e^2/h$ for the system containing two spins and two valleys.

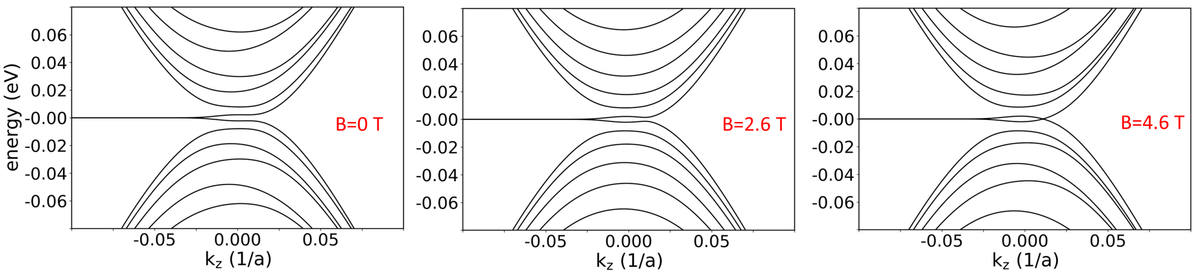

Figure 7: The energy bands with different applied magnetic fields are presented for the arclength 150 nm carbon nanoscrolls.

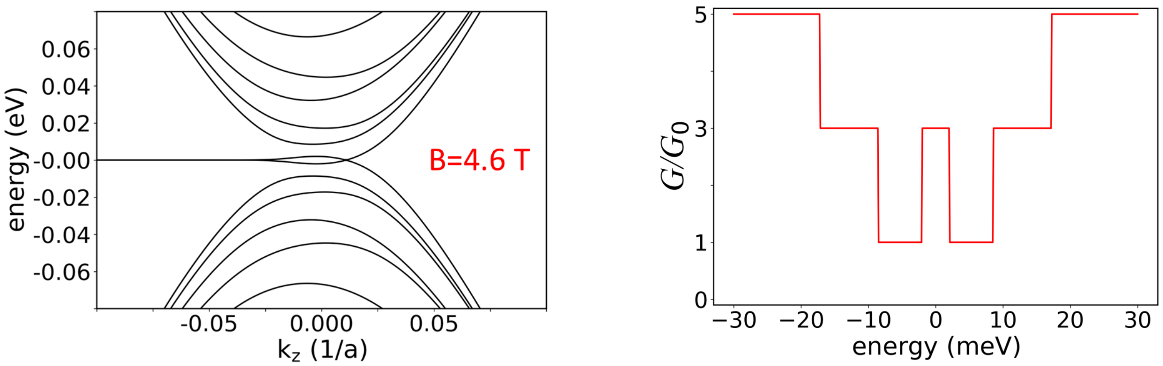

Figure 8: The energy bands with magnetic field 4.6 Tesla and the conductance are illustrated for the arclength 150 nm carbon nanoscroll.

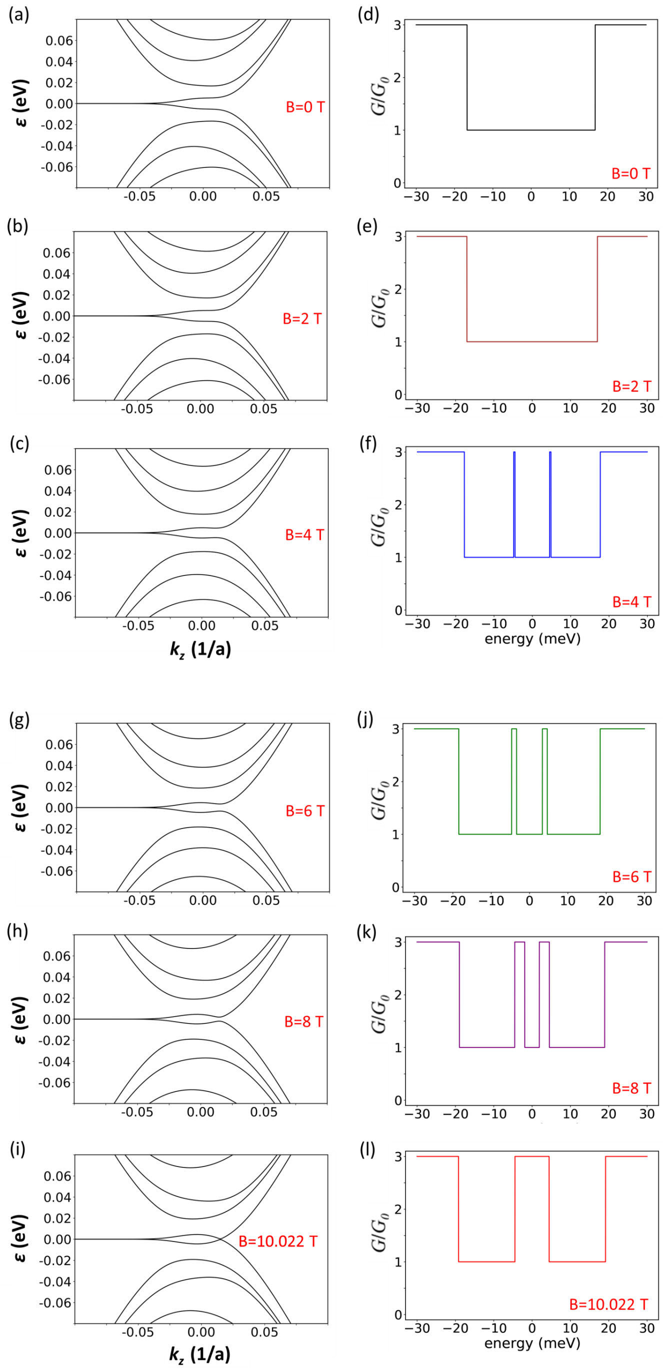

Figure 9: Conductance varies with the strength of the applied magnetic field. The left panel illustrates the energy bands under different applied fields, while the right panel shows the conductance corresponding to these magnetic field strengths.

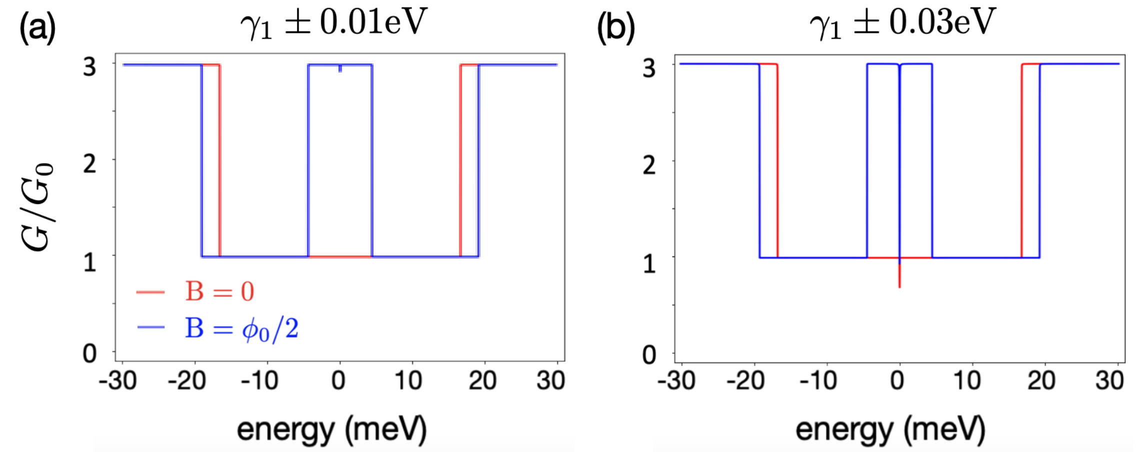

Figure 10: Conductance as a function of energy for the two-turn nanoscroll (a) with tiny random variation (0.381 ± 0.01 eV) and (b) with remarkable random variation (0.381 ± 0.03 eV) in the interlayer hopping parameter. The blue line is the conductance in magnetic field and the red line is the conductance without magnetic field.

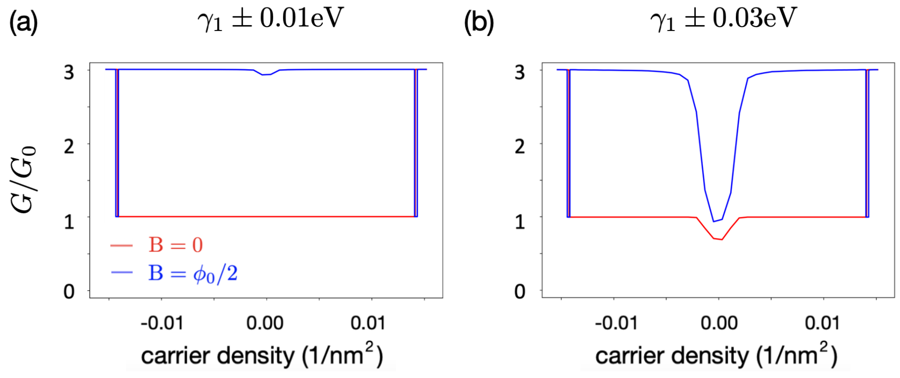

Figure 11: Conductance as a function of carrier density for the two-turn nanoscroll (a) with tiny random variation (0.381 ± 0.01 eV) and (b) with remarkable random variation (0.381 ± 0.03 eV) in the interlayer hopping parameter. The blue line is the conductance in magnetic field and the red line is the conductance without magnetic field.

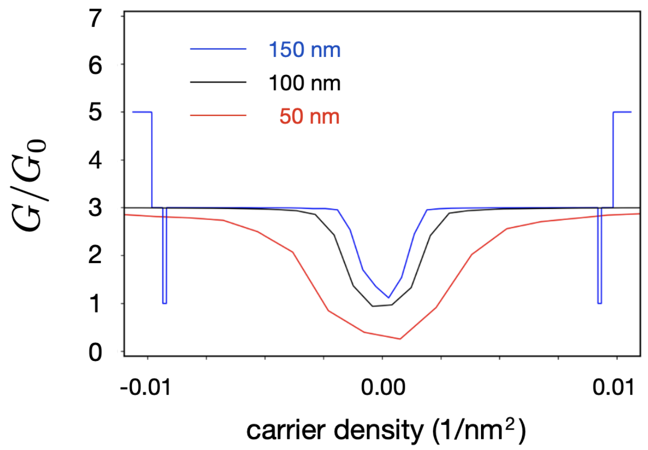

Figure 12: Conductance as a function of carrier density for the two-turn nanoscroll in the magnetic flux and with remarkable random variation (0.381 ± 0.03 eV) in the interlayer hopping parameter

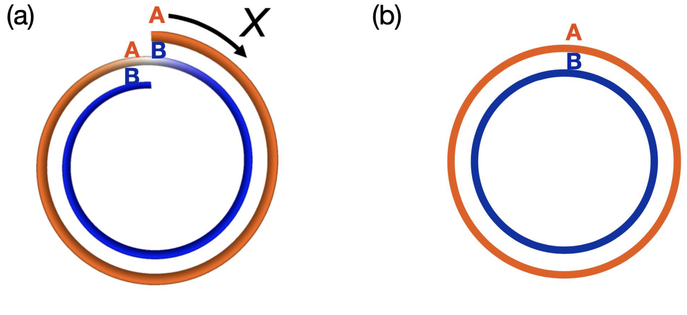

Figure 13: The two-turn nanoscroll and two-turn nanotube based on connecting the opened boundaries of the AB-stacked bilayer graphene ribbon.

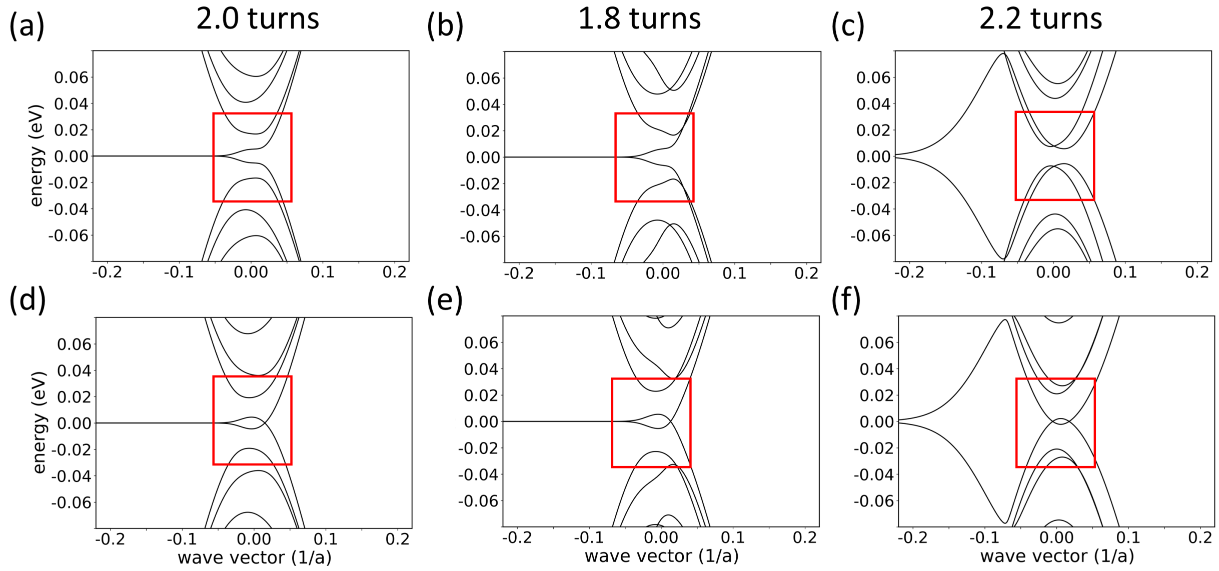

Figure 14: Band structure of the (a) 2.0- (b) 1.8-, and (c) 2.2-turns nanoscroll with a fixed arclength $X = 100$ nm. Panel (d), (e) and (f) are the bands of the same nanoscroll but with applied magnetic flux. Dashed boxes mark the approximate energy range of bands contributing to the magnetotransport in our study.

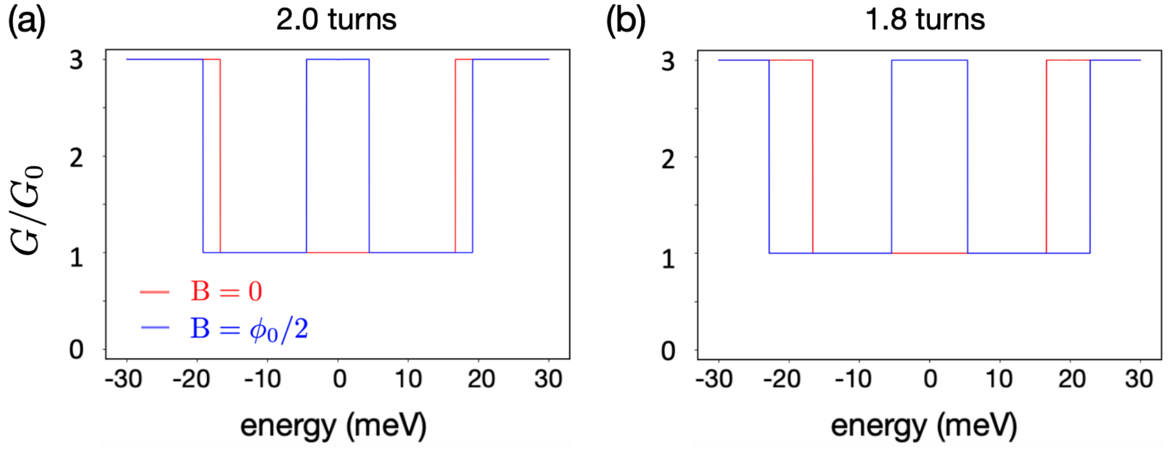

Figure 15: The conductance of 2 and 1.8 turns CNSs are provided in panels (a) and (b), respectively. The blue (red) line denotes the system with (without) applied magnetic flux.
